# Organizational performance impacting patient satisfaction in Ontario hospitals: a multilevel analysis

**DOI:** 10.1186/1756-0500-6-509

**Published:** 2013-12-05

**Authors:** Anna J Koné Péfoyo, Walter P Wodchis

**Affiliations:** 1 Institute of Health Policy, Management and Evaluation, Faculty of Medicine, University of Toronto, 155 College St, 4th floor, Toronto, ON M5T 3 M6, Canada; 2 Cancer Care Ontario, Toronto, Canada; 3 Toronto Rehabilitation Institute, Toronto, Canada; 4 Institute for Clinical Evaluative Sciences, Toronto, Canada

**Keywords:** Patient satisfaction, Experience of care, Organizational performance, Determinants of satisfaction, Quality of care, Multilevel model

## Abstract

**Background:**

Patient satisfaction in health care constitutes an important component of organizational performance in the hospital setting. Satisfaction measures have been developed and used to evaluate and improve hospital performance, quality of care and physician practice. In order to direct improvement strategies, it is necessary to evaluate both individual and organizational factors that can impact patients’ perception of care. The study aims were to determine the dimensions of patient satisfaction, and to analyze the individual and organizational determinants of satisfaction dimensions in hospitals.

**Methods:**

We used patient and hospital survey data as well as administrative data collected for a 2008 public hospital report in Ontario, Canada. We evaluated the clustering of patient survey items with exploratory factor analysis and derived plausible dimensions of satisfaction. A two-level multivariate model was fitted to analyze the determinants of satisfaction.

**Results:**

We found eight satisfaction factors, with acceptable to good level of loadings and good reliability. More than 95% of variation in patient satisfaction scores was attributable to patient-level variation, with less than 5% attributable to hospital-level variation. The hierarchical models explain 5 to 17% of variation at the patient level and up to 52% of variation between hospitals. Individual patient characteristics had the strongest association with all dimensions of satisfaction. Few organizational performance indicators are associated with patient satisfaction and significant determinants differ according to the satisfaction dimension.

**Conclusions:**

The research findings highlight the importance of adjusting for both patient-level and organization-level characteristics when evaluating patient satisfaction. Better understanding and measurement of organization-level activities and processes associated with patient satisfaction could contribute to improved satisfaction ratings and care quality.

## Background

Hospitals that are committed to providing excellent health care outcomes, including patient satisfaction, establish multiple programs and initiatives to achieve these goals. Measuring performance is essential to assessing the effects of continuous efforts to improve quality of care and ensuring the pursuit of excellence in hospitals [1]. Increasingly, patient satisfaction is acknowledged as a key organizational performance measure [2-4]. In addition to providing a unique perspective on the performance of a hospital, patient satisfaction is considered as a predictor of a patient’s willingness to follow treatment, to return for service, or to recommend a service to others [5-7]. In the US, the Centers for Medicare and Medicaid Services (CMS) has identified patient experience of care as an important determinant of the performance payments to be made to acute hospitals [8]. Thus, from fiscal year 2013 at least 0.3% of hospitals’ Medicare revenue (30% of the 1% withhold from participating hospitals’ Diagnosis-Related Group payments) will be determined by their performance on these experience/satisfaction measures [9].

Patient experience and satisfaction measures have been developed and used to evaluate and improve hospital performance, quality of care and physician practice [10-12]. Many studies have analyzed determinants of satisfaction at the individual level. Factors associated with patient satisfaction include socio-demographic characteristics, expectations, health status, patient–provider relationship, facility setting, and urgency of admission, among others [13-17]. However, variations among organizations/countries remain, even after taking into account individual characteristics [13]. Some authors have evaluated the impact on patient satisfaction of organizational factors, including service climate, nurse performance, facility/clinic size, number of beds, physical amenities, or access to electronic records [18-20]. As mentioned by Kazley et al [21] or Greenslade and Jimmieson [18], a void exists in the literature about the relationship between patient satisfaction and organizational aspects like electronic health records or service climate. To understand the determinants of patient experience and satisfaction, it is necessary to consider both individual and organizational level factors that impact patients’ perception of the care they receive. Patient-level factors are important, as they provide information about the circumstances of the individual’s encounter with the health system; however, the most important organizational level factors are those that are modifiable by health care organizations and represent opportunities to improve patient satisfaction and quality of care.

The purpose of this study was first, to validate the satisfaction questionnaire and relevant dimensions of patient satisfaction in the Ontarian population and second, to measure specific determinants of satisfaction in each dimension at both individual patient and organization levels. Although Ontario has used the National Research Corporation Canada (NRCC) (formerly NRC Picker Canada) Patient Satisfaction Survey to report to the public on hospital quality since 2003, the instrument has not been validated in the Canadian context. Moreover, organizational determinants of satisfaction have not been examined. Using a multilevel approach to detect not only patient characteristics but also organizational factors associated with satisfaction provides a more comprehensive understanding of satisfaction than analyzing only one level of determinants.

## Methods

### Study population

This study included data from the 2008 Ontario Hospital Report. Data were collected between April 1, 2006, and March 31, 2007, including measures of patient satisfaction, hospital internal business processes, financial performance, and clinical outcomes. The 2008 Ontario Hospital Report included data measuring patient satisfaction based on 54,760 survey responses (in 83 hospitals), with a response rate of 42% (from 130,400 sampled individuals) [22].

A hospital-level survey was used to measure internal business processes in 103 facilities. Financial performance was measured using mandatory standardized financial and statistical reporting data and clinical outcomes were measured using a mandatory clinical administrative database available to all Ontario hospitals. The final sample of 68 hospitals is based on those that completed both inpatient satisfaction and internal business process surveys. The characteristics of patients and hospitals in this final sample did not substantially differ from the initial sample, apart from the proportion of small hospitals – 16% of all participating hospitals versus 9% in the final sample – as small hospitals were less likely to provide complete responses in the satisfaction surveys. The data represent the most recent year with all four data sources.

Ethics approval was not sought for this study, as it did not involve sensitive personal health information. The information used was available for research purposes at the Health System Performance Research Network (HSPRN), University of Toronto, and included mandatory administrative data gathered for the Ontario Hospital Reports. Patients’ consent for the Satisfaction Survey was obtained with the assurance of data confidentiality.

### Measures

The NRCC acute care inpatient satisfaction survey was adopted for the Ontario Hospital Report beginning in 2004. This questionnaire measures patients’ perception of the care they receive and is distributed to a sample of hospital patients 6 to 12 weeks after their inpatient stay. Following exploratory factor analyses to determine appropriate constructs of patient satisfaction in our observed sample, measures of patient satisfaction were estimated by average scores on questionnaire items within each factor. Satisfaction was measured at the individual patient level using these data.

To evaluate the association between patient satisfaction and financial performance, we included in our analysis four of the eight financial performance indicators used in the Ontario Hospital Report 2008. The four indicators retained were financial viability (total margin), capital (% equipment expenses) and human resources (% sick time, % registered nurses). Table 1 describes the operationalization of these indicators. Percentage of corporate services, current ratio, debt service coverage, and unit cost performance were not included due to lack of relevance in the literature and also for the sake of brevity. Moreover, these indicators have some operationalization and interpretation issues.

**Table 1:** Areas of interest related to organizational performance

**Areas**	**Selected questions/Definition**	**Variables derived**
**Financial viability**	The percent by which a hospital's total revenue differs from its total expenses, excluding the impact of facility amortization (land, building and building service equipment). (Revenue – Expenses) * 100 / Revenues	Total margin
**Capital**	How much a hospital spends in a given year to operate and maintain its computer systems, x-ray machines, and other capital equipment, compared to its total expenses. Equipment expense * 100 / Expenses	% Equipment expenses
**Human resources**	The proportion of full-time patient care personnel hours that were paid sick hours Sick hours * 100 / Full-time earned hours	% Sick time
	The proportion of nursing care hours that were provided by registered nurses Acute inpatient registered nursing earned hours * 100 / Acute inpatient nursing earned hours	% Registered nurses
	Staff Roles: Which of the following staff roles currently exist in your organization? (Nurse practitioner, Nurse specialist, Nurse educator in ED, Staff for professional practice, Clinical specialist, Hospitalist, Social worker, Case manager, Staff for physician recruitment, Volunteer coordinator, Decision support role, Telehealth coordinator, Utilization review analyst, Risk management analyst, Staff for equity issues, Ombudsperson)	Number of permanent roles
**Professional development and learning**	What percentage of physicians with administrative roles participated in continuing education activities (e.g., formal in-service programs, internal/external courses and conferences) supported by your organization	Some or most professionals are educated in quality
	What percentage of nursing staff participated in continuing education activities (e.g., formal in-service programs, internal/external courses and conferences) supported by your organization?	
	What percentage of other patient care staff participated in continuing education activities (e.g., formal in-service programs, internal/external courses and conferences) supported by your organization?	
**Use of information technology**	Are electronic records and data currently being used in your organization as a primary source of information? (Patient visit registration information; Diagnostic imaging reports; Electronic medical images; Diagnostic laboratory results; Patient-based pharmacy/drug profiles; Nursing and physician clinical documentation; Clinical documentation by other health professionals)	Number of areas where electronic records and date are used
**Use and dissemination of information for clinical decision-making**	For each of the following clinical measures, to what extent are these data currently collected, shared and used in your organization? (Unplanned return to the OR; Hospital-acquired infection or sepsis; Adverse drug reaction; Unplanned injury or unplanned repair of organ during surgery; Unplanned transfer to ICU/CCU; In-hospital mortality; Hospital-acquired injury (e.g., falls); Waiting time to gain access from the ED to inpatient bed; Unplanned readmission; Percent of day-surgery patients; Percent procedures completed on scheduled day)	Number of areas where data are collected and shared
**Patient safety**	Our organization has a policy for hand hygiene.	Yes/No
	Our organization has adopted patient safety as a written, strategic priority/goal.	Yes/No
	Our organization has implemented a formal policy and process of disclosure of adverse events to patients/families, including support mechanisms for patients, family, and care/service providers.	Yes/No
**Reconciling medications across the continuum of care**	Patient’s current medications are reconciled against those prescribed in hospital on admission and with the patient’s involvement.	Yes/No
	A complete list of the patient's medications is reconciled and communicated to the next provider of health care service when the patient is referred or transferred to another setting, service, practitioner, or level of care within or outside of the hospital.	Yes/No

In order to measure the internal business processes, we reviewed sections of the survey developed for the Ontario Hospital Report that included the following categories: management of human resources, use of information technology, use and dissemination of information for clinical decision making, use and dissemination of information for quality improvement, healthy work environment, and patient safety. Based on the literature, six areas were retained as potential determinants of patient satisfaction (Table 1): staff roles (in human resources), professional development and learning, use of information technology, use and dissemination of information for clinical decision-making, patient safety, and reconciling medications across the continuum of care).

Other patient-level demographic factors (age, sex, education level) and clinical measures (length of stay; number of hospital stays in prior six months; perceived health; planned or unplanned, i.e., urgent, admission; experience of pain) were included in the analysis. Finally, hospital-level clinical performance measures of risk-adjusted medical adverse events (post-admission pressure ulcers, fractures from falls, and pneumonia associated with acute myocardial infarction or AMI, heart failure, asthma, gastrointestinal or GI bleed, and stroke) and readmissions were included.

### Analysis

Exploratory factor analysis was used to evaluate the clustering of items included in the questionnaire and to derive dimensions of satisfaction for analysis (results not shown). Principal factor analysis (PFA) was first applied to all items in order to identify the underlying dimensions from the variance common to the items. A separate analysis for each derived factor was then performed. Commonalities, percentage of variance explained, and regression coefficients were analyzed. Reliability of factors was assessed with Cronbach’s Alpha.

A total of 43 questionnaire items were included in the analysis. All item responses were measured on an ordinal scale (e.g., not at all, sometimes, always) and polychoric correlations (appropriated for non-continuous responses) were used.

After deriving latent factors from the items, we calculated average scores for each factor. Dimension scores were calculated conditional on valid response values for at least two-thirds of the items if the factor had more than five items, and all items if the factor had five or less. To analyze the criterion-related and convergent/discriminant validity of satisfaction dimensions, relationships between the dimensions and with socio-demographic characteristics of patients were tested with ANOVA tests or Pearson correlations. The average scores of satisfaction were rescaled between 0% (minimum) and 100% (maximum) to facilitate interpretations and to ensure that the same scale was applied for all factors.

A hierarchical multivariate model was fitted to evaluate the determinants of satisfaction. Specifically we evaluated the associations between each satisfaction domain and measures of hospital organizational performance from the Ontario Hospital Report, accounting for individual patient characteristics. Measures at two levels were considered. Patient satisfaction ratings and characteristics were measured at the patient level and nested within hospitals; hospital performance was measured at the organization level. Regression models included data from 68 institutions, including between 71 and 5,027 patient respondents for each hospital. Because patient surveys were not always completed, the range of respondents varied by domain. No patient scores were imputed but all of the available data (list-wise deletion) were used; therefore, the number of observations in each model varies. In order to evaluate the distribution of variance between levels while analyzing the contribution of each level’s characteristics, we started by fitting null models including only random intercepts and no explanatory variables. Then we fitted final mixed models including random effects and all characteristics’ effects. We also examined the model variances explained with the initial null model with only patient characteristics, and subsequently with both patient and hospital characteristics.

Analyses were done with SPSS (PASW Statistics version 18) and MLwiN version 2.20 software. Polychoric correlations were computed with R packages (PSYCH and POLYCOR).

## Results

Table 2 provides a description of patient characteristics and organizational measures. The average age of respondents was 63 years (±16); 49.7% were males and 20.8% had a university degree; and 14.2% of questionnaires were completed by someone other than the patient (e.g., spouse, child, caregiver). Nearly eight percent (7.9%) reported their health status as excellent and 30.7% as poor or fair. Most respondents reported more than one hospitalization during the six months prior to completing the survey (69.7%). The most frequent admission type was through an emergency department (ED) (49.1%), followed by planned admission (40.3%). Survey responses were proportional to the size of hospital. Just 2% of the surveys came from patients treated in small hospitals, while 53% were discharged from a community setting and 45% from a teaching hospital. Satisfaction data are drawn from 6 small hospitals, 49 community hospitals and 13 teaching hospitals, all of which participated in the organization survey as well. Readmissions represented on average 4.1% of hospital discharges and 81.9% of the nursing staff complement was registered nurses (RNs). Almost all hospitals reported having a policy for hand hygiene (92.6%) or had adopted patient safety as a written, strategic priority (88.2%). Approximately 20% reported that a list of the patient's medications was reconciled against those prescribed in hospital on admission or a complete list of the patient's medications was reconciled and communicated to the next provider.

**Table 2:** Description of Patient and Organizational characteristics (37 440 patients in 68 hospitals)

**Patients characteristics**		**% or Mean ± SD**
**Age in decade (mean ± SD)**		6.3 ± 1.6
**Sex**	Male	49.7
**Length of stay (mean ± SD)**		6.7 ± 9.6
**Hospital stay**	Only this time	11.1
	This time and one other time	19.1
	This time and more than one other	69.7
**Education level**	Public or high school	52.8
	College, trade, or technical school	26.3
	University	20.8
**Someone else filled questionnaire**		14.2
**Perceived health**	Poor or fair	30.7
	Good	35.7
	Very good	25.7
	Excellent	7.9
**Admission**	Emergency	49.1
	Planned admission	40.3
	Transferred from another facility	6.1
	Other unplanned admission	4.6
**Pain**	Yes	70.7
**Hospitals characteristics**		**% or Mean ± SD**
**Peer group**	Small	8.8%
	Community	72.1%
	Teaching	19.1%
**Medical nurse-sensitive adverse events (in%)**		0.9 ± 0.7
**Medical readmissions (in%)**		4.1 ± 2.7
**Total margin**		1.1 ± 2.6
**Equipment expenses (in%)**		6.7 ± 1.3
**Registered nurses (in%)**		81.6 ± 11.8
**Percentage of sick time**		4.4 ± 0.8
**Number of permanent staff roles**		11.0 ± 3.6
**Some or most professionals participated in continuing education activities about quality**		69.1%
**Number of areas where electronic records and data are used**		5.7 ± 1.5
**Number of areas where data are collected and shared**		9.2 ± 2.4
**Policy for hand hygiene (Yes)**		92.6%
**Adopted patient safety as a written, strategic priority/goal (Yes)**		88.2%
**Formal policy and process of disclosure of adverse events to patients/families (Yes)**		64.7%
**Patient’s current medications reconciled against those prescribed in hospital on admission and with the patient’s involvement. (Yes)**		19.1%
**Complete list of the patient's medications reconciled and communicated to the next provider when the patient is referred or transferred (Yes)**		20.6%
**Patient satisfaction**		**Mean ± SD**
**Overall rating- Nurses and doctors**		74.5 ± 20.5
**Overall rating- Patient-centered care**		64.7 ± 15.5
**Admission process**		79.6 ± 26.7
**Availability of staff**		84.0 ± 20.7
**Communication with patient**		81.5 ± 18.3
**Communication with family**		80.6 ± 25.7
**Discharge transition**		70.7 ± 30.7
**Pain management**		76.4 ± 15.8

### Satisfaction dimensions and validity

We found eight satisfaction dimensions that had item sets with acceptable to good level of loadings and commonalities as well as good reliability of the scales. The dimensions are: Nurses and doctors (7 items, alpha = 0.95); Patient-centered care (7 items, alpha = 0.82); Admission process (3 items, alpha = 0.88); Availability of staff (3 items, alpha = 0.80); Communication with patients (11 items, alpha = 0.87); Communication with family (4 items, alpha = 0.76); Discharge transition (4 items, alpha = 0.71) and Pain management (4 items, alpha = 0.60). A descriptive analysis of the satisfaction dimensions is shown in Table 2. The highest rating was for staff availability and the lowest was for patient-centered care.

All dimensions of satisfaction were positively and significantly correlated, which means satisfaction in one dimension is associated with satisfaction in other dimensions (results not shown). However, some correlations were small and pain management showed the smallest correlations with other dimensions.

### Determinants of patient satisfaction and association with hospitals’ performance

All individual characteristics were statistically associated with each dimension of patient satisfaction (Table 3). However, some differences were more meaningful (10 percentage points or more) than others. Satisfaction ratings for each additional age decade increased by 0.8 percentage points (Availability of staff) and 1.9 (Communication with family); for each additional day in hospital, a decrease of between 0.03 and 0.17 in satisfaction ratings was found. Perceived health and type of admission showed the most significant associations. People in excellent health rated their doctors 12.9 percentage points higher and Discharge Transition 10.6 percentage points higher, compared to people with low selfrated health. Patients with planned admissions had satisfaction scores with the Admission Process that were 20.3 percentage points higher than those admitted through an ED.

**Table 3:** Multilevel regression of determinants of patient satisfaction (37 440 patients in 68 organizations)

	**Nurses & doctors**	**Patient-centered care**	**Admission process**	**Availability of staff**	**Communication with patient**	**Communication with family**	**Discharge transition**	**Pain management**
**Number of respondents**	**37231**	**37339**	**33267**	**19993**	**33700**	**21719**	**24832**	**24108**
**Fixed effects**	Coef (s.e.)	Coef (s.e.)	Coef (s.e.)	Coef (s.e.)	Coef (s.e.)	Coef (s.e.)	Coef (s.e.)	Coef (s.e.)
Patient level								
Constant	**62.2 (6.4)**	**55.9 (4.8)**	**72.7 (8.1)**	**86.4 (6.8)**	**74.6 (5.2)**	**56.8 (6.7)**	**59.7 (8.3)**	**65.3 (3.6)**
Age in decade	**0.9 (0.1)**	**1.1 (0.1)**	**1.3 (0.1)**	**0.8 (0.1)**	**1 (0.1)**	**1.9 (0.1)**	0 (0.1)	**1.2 (0.1)**
Male	**2.4 (0.2)**	**2.7 (0.2)**	**2.3 (0.3)**	**3.6 (0.3)**	**1.5 (0.2)**	**3.6 (0.3)**	**7.1 (0.4)**	**1.6 (0.2)**
Length of stay	**-0.03 (0.01)**	**-0.06 (0.01)**	0 (0.01)	**-0.14 (0.01)**	**-0.08 (0.01)**	-0.04 (0.02)	**-0.17 (0.02)**	**-0.03 (0.01)**
Hospital stay (ref = Only this time)								
This time and one other time	0.7 (0.4)	**1 (0.3)**	**1.7 (0.5)**	**1.9 (0.5)**	**1.8 (0.4)**	1 (0.6)	1 (0.7)	**1.9 (0.4)**
This time and more than one other	**1.3 (0.3)**	**1.6 (0.3)**	**3.4 (0.4)**	**3.4 (0.4)**	**3.1 (0.3)**	**1.3 (0.5)**	**1.3 (0.6)**	**2.6 (0.3)**
Education level (ref = Public or high school)								
College, trade, or technical school	**-2 (0.2)**	**-2 (0.2)**	**-1 (0.3)**	**-2.2 (0.3)**	**-1.1 (0.2)**	**-3 (0.4)**	**-3.8 (0.5)**	**-1.7 (0.2)**
University	**-4.1 (0.3)**	**-3.6 (0.2)**	**-2.6 (0.4)**	**-4.8 (0.4)**	**-2.4 (0.3)**	**-5.1 (0.5)**	**-7.1 (0.5)**	**-2.6 (0.3)**
Someone else filled the questionnaire (ref = Patient)	**-5.8 (0.3)**	**-3.7 (0.2)**	**-6.3 (0.4)**	**-7.4 (0.4)**	**-5.3 (0.3)**	**-2.9 (0.5)**	**-2.4 (0.6)**	**-3.4 (0.3)**
Perceived health (ref = Poor)								
Good	**3.7 (0.3)**	**2.8 (0.2)**	**3.2 (0.3)**	**3 (0.4)**	**3.4 (0.2)**	**3.5 (0.4)**	**5.4 (0.5)**	**2.7 (0.3)**
Very Good	**9.9 (0.3)**	**6.7 (0.2)**	**5.5 (0.4)**	**5.8 (0.4)**	**7 (0.3)**	**7.6 (0.5)**	**10.1 (0.6)**	**4.5 (0.3)**
Excellent	**12.9 (0.4)**	**8.1 (0.3)**	**6.4 (0.6)**	**4.9 (0.6)**	**7.7 (0.4)**	**8.5 (0.7)**	**10.6 (0.8)**	**4.4 (0.4)**
Admission (ref = Emergency)								
Planned admission	**5.6 (0.2)**	**2.5 (0.2)**	**20.3 (0.3)**	**2.3 (0.3)**	**5.5 (0.2)**	**5.8 (0.4)**	**9.3 (0.4)**	**3.8 (0.2)**
Transferred from another facility	**5.3 (0.4)**	**4 (0.3)**	**19.6 (0.6)**	**3.5 (0.6)**	**3.5 (0.4)**	**1.6 (0.7)**	**3.6 (0.8)**	**3.6 (0.4)**
Other unplanned	**4.5 (0.5)**	**2.1 (0.4)**	**10.5 (0.6)**	**3.4 (0.7)**	**3.1 (0.5)**	**5.2 (0.8)**	**7.4 (0.9)**	**2.8 (0.5)**
No pain (ref = Yes)	**3 (0.2)**	**3.1 (0.2)**	**1.7 (0.3)**	**3 (0.3)**	**2.7 (0.2)**	**2.7 (0.4)**	**1.6 (0.4)**	N/A
Hospital level								
Peer group (ref = Community)								
Small	1.8 (2.4)	**4.3 (1.8)**	**6.1 (3)**	-0.4 (2.6)	0.7 (2)	4.2 (2.7)	2.9 (3.2)	1.5 (1.4)
Teaching	2.1 (1.4)	1.6 (1)	1.6 (1.8)	1.8 (1.4)	1.1 (1.1)	1.6 (1.4)	2.6 (1.7)	0.5 (0.7)
Medical nurse-sensitive adverse events (%)	-1.7 (0.9)	**-1.5 (0.6)**	-2.1 (1.1)	**-2.1 (0.9)**	-1.3 (0.7)	-0.5 (0.9)	-1.6 (1.1)	-0.4 (0.5)
Medical readmissions (%)	0.2 (0.2)	0.1 (0.2)	0.3 (0.3)	0.2 (0.2)	0.1 (0.2)	0.3 (0.3)	0.3 (0.3)	-0.1 (0.1)
Total margin	-0.3 (0.2)	-0.1 (0.2)	-0.3 (0.3)	-0.1 (0.2)	-0.1 (0.2)	-0.1 (0.2)	-0.3 (0.3)	0.1 (0.1)
Equipment expenses	0.4 (0.4)	**0.7 (0.3)**	0.8 (0.6)	0.1 (0.5)	0.1 (0.4)	0.5 (0.5)	0.4 (0.6)	0 (0.3)
Registered nurses (%)	-0.1 (0.1)	-0.1 (0.1)	**-0.2 (0.1)**	**-0.2 (0.1)**	-0.1 (0.1)	0 (0.1)	0 (0.1)	0 (0)
Percentage of sick time	**-1.5 (0.7)**	**-1.6 (0.6)**	-1.6 (0.9)	-1.3 (0.8)	-0.8 (0.6)	-0.7 (0.8)	-0.9 (1)	-0.4 (0.4)
Number of permanent staff roles	-0.1 (0.2)	-0.1 (0.2)	-0.3 (0.3)	-0.1 (0.2)	-0.1 (0.2)	-0.1 (0.2)	-0.3 (0.3)	-0.1 (0.1)
Continuing education about quality	1.3 (1.1)	1.1 (0.8)	0.7 (1.4)	-1.1 (1.2)	0.5 (0.9)	2.1 (1.2)	1 (1.4)	-0.3 (0.6)
# areas where e-records and data are used	0.7 (0.4)	**0.6 (0.3)**	0.7 (0.5)	0.6 (0.5)	0.4 (0.4)	0.5 (0.4)	0.8 (0.5)	0.1 (0.2)
# areas where data are collected and shared	0.2 (0.2)	0.1 (0.2)	0.3 (0.3)	0.2 (0.3)	0 (0.2)	-0.2 (0.3)	-0.2 (0.3)	**0.2 (0.1)**
Policy for hand hygiene	**4 (1.9)**	2 (1.4)	1.1 (2.4)	3.2 (2)	2.8 (1.6)	1 (2)	1.4 (2.4)	1.4 (1)
Patient safety as a written, strategic priority.	1 (1.7)	1.4 (1.3)	-0.4 (2.2)	3.2 (1.9)	0.7 (1.4)	1.5 (1.9)	1 (2.3)	0 (1)
Formal policy and process of disclosure of adverse events to patients/families	0.6 (1.1)	0.7 (0.8)	2.2 (1.4)	1.7 (1.1)	0.6 (0.9)	0 (1.1)	1.6 (1.4)	**1.3 (0.6)**
Patient’s current medications reconciled on admission and with patient’s involvement.	-0.5 (1.4)	-0.3 (1.1)	-1.9 (1.8)	-2.2 (1.5)	-0.7 (1.2)	1.8 (1.5)	0 (1.8)	-1 (0.8)
Patient's medications reconciled and communicated to next provider	2.3 (1.4)	**2.3 (1.1)**	3.3 (1.8)	**3.5 (1.5)**	2.3 (1.2)	0.7 (1.5)	2.2 (1.9)	**1.7 (0.8)**

In bold: p < 0.05.

Few organizational performance indicators were associated either positively or negatively with patient satisfaction. Type of Hospital did not have a significant impact on patient satisfaction, apart from satisfaction with patient-centered care and the admission process, both of which were higher among small hospitals. Patients in small hospitals rated their experience on average 4.3 (Patient-centered care) and 6.1 (Admission process) percentage points higher compared to those in community hospitals (Table 3). As an example, a one point increase in the rate of adverse events among medical patients or the proportion of sick time contributed to a decrease in satisfaction ratings of 1.5 percentage points or more.

The final hierarchical models explained 5 to 17% of variation at the patient level and up to 52% of variation between hospitals (Table 4). Variances at individual and hospital levels were statistically significant but intraclass correlations were small in both null and final mixed models, which means differences in satisfaction scores are mainly at the individual level. In fact, variance at the hospital level represented between 2% and 6% of the total variance for each satisfaction measure (Table 4). For example, in satisfaction with nurses and doctors, the null model identifies that the variance between hospitals accounted for 4.2% of the total variance, with the remaining 95.8% attributable to between-patient variance. In the final model, the remaining unexplained variance at the hospital level represented 3% of the total remaining unexplained variance.

**Table 4:** Hierarchical modeling statistics

	**Overall rating-nurses & doctors**	**Overall rating- patient-centered care**	**Admission process**	**Availability of staff**	**Communication with patient**	**Communication with family**	**Discharge transition**	**Pain management**
**Variance estimates**								
**Null model**								
Patient level	405.0	230.1	682.8	409.1	324.3	649.9	921.6	245.8
Hospital level	17.6	13.5	38.3	22.8	11.6	12.7	23.0	3.8
**Intermediate model (patient measures only)**								
Patient level	365.0	209.5	566.0	379.3	295.1	616.8	857.9	231.8
Hospital level	14.5	12.3	39.0	18.9	9.9	10.3	17.7	3.0
**Final model (patient and hospital measures)**								
Patient level	365.0	209.5	565.9	379.3	295.1	616.7	857.9	231.8
Hospital level	11.4	6.5	18.3	11.9	7.3	9.9	16.2	2.6
**% of Variance explained compared to null model**								
**Intermediate model (patients measures only)**								
Patient level	11%	10%	17%	8.0%	10%	5.0%	7.0%	6.0%
Hospital level	18%	9.0%	-2.0%	17.0%	14%	19%	23%	21%
**Final model (patients and hospitals measures)**								
Patient level	11%	10%	17%	8.0%	10%	5.0%	7.0%	6.0%
Hospital level	35%	52%	52%	48%	37%	22%	29%	32%
**Intraclass correlations**								
Null model	4.2%	5.5%	5.3%	5.3%	3.5%	1.9%	2.4%	1.5%
Intermediate model (patient measures only)	3.8%	5.5%	6.4%	4.7%	3.2%	1.6%	2.0%	1.3%
Final model (patient and hospital measures)	3.0%	3.0%	3.1%	3.0%	2.4%	1.6%	1.9%	1.1%

## Discussion

### Satisfaction dimensions

The topics and items included in the satisfaction questionnaire came from research by the Picker Institute, including a literature review, structured focus groups with patients, families, and health professionals, as well as pilot interviews followed by critiques from patients and health professionals. The purpose was to identify issues important to patients [23]. Because the survey instrument had not previously been validated in the Ontarian population, it was necessary to identify meaningful, valid, and reliable areas of satisfaction and ensure that the analyses in this study focused on valid outcomes.

The dimensions that were empirically identified in this study varied from Picker’s Eight Dimensions of Patient-centred Care and from what has been proposed by other satisfaction research [24]. For example, in this study a new, stand-alone factor related to pain management that was relevant to Ontario patients was identified. Also, items related to access and timeliness, identified as a separate domain in Picker’s dimensions, were found to be related to patients’ ratings of their doctors and nurses and to communication. The questionnaire has good validity based on the factors derived. Reliability of the estimated scales were quite good. The satisfaction scores also varied according to patients’ socio-demographic characteristics, providing evidence of the discriminant validity of the measurement tool.

### Factors associated with satisfaction

The most variation in patient satisfaction ratings was found at the individual level rather than at the hospital level, as shown by intraclass correlations. This suggests that hospitals have relatively little opportunity to substantially change patient satisfaction scores and that it is important to adjust for differences in (non-modifiable) patient characteristics when comparing satisfaction between hospitals. This is consistent with previous research in primary care that used a three-level regression (patient, physician, practice) to examine associations with four satisfaction outcomes and found a very small proportion of the variation at practice and physician levels [25]. Although satisfaction was more strongly associated with patient characteristics than between-hospital variation, the inclusion of organizational factors did significantly increase explained variance for all satisfaction dimensions, except for discharge transition and communication with family, with most associations in the expected direction. Between 22 and 52% of the between-hospital variation was explained using these organizational variables, demonstrating that the variables included are important determinants of hospital performance.

Overall, patients suffering from more severe illnesses (poor perceived health, functional limitation as described by respondents, and admission through an ED) were less satisfied and gave lower ratings on their experience. These patients may require more attention and, with additional burden and pressure on the hospital, may be less likely to receive sufficient attention. The findings of this study show that efficient planning at the admission stage could make an important difference to patient satisfaction and facilitate a smooth admission process. This could also be indicative of further challenges that need to be overcome in EDs, which could impact the quality of care offered to patients. Longer stays potentially arising from more serious health conditions, post-admission complications, and/or poor discharge planning were related to lower satisfaction for all satisfaction measures except Admission Process. The lack of a relationship between length of stay and the admission process provides a useful counterfactual, because admission occurs before patients know how long they will spend in the hospital.

The reasons for lower ratings of care among respondents with more education can only be speculated upon. The result may reflect that these individuals have either higher expectations or are more critical in their evaluation of care. Patients with higher education may be more knowledgeable about their condition and have higher expectations about their involvement in decision-making and care processes. In this engagement, interpersonal tensions or unmet expectations of staff-provider interaction might lead to lower ratings of their care experience. Surveys completed by someone other than the patient also had lower ratings. This might reflect unfavourable experiences among patients in worse condition or that caregivers are more critical about the experience compared to the patients themselves.

Organizational performance indicators do play a role in patient satisfaction and therefore quality of care. Dimensions of hospital organizational performance were associated with related dimensions of patient satisfaction. Aspects of patient safety, like disclosure of adverse events to patients and family or effective management of patients’ medications, were a significant factor for satisfaction in pain management. These patient-centered practices could contribute to facilitating interactions with patients and families and helping to achieve better processes. Corroborating evidence was reported by O’Connor and Shewchuk [26]. Unsurprisingly, a higher rate of adverse events was associated with lower patient perceptions of the quality of patient-centered care offered and lower ratings of availability of staff, or nurses and doctors. Higher ratings of hospitals’ patient-centered care and admission process were found among patients in small hospitals, unlike Klinkenberg and colleagues [27], who found higher satisfaction for patients at larger hospitals (200 or more beds). The latter study only examined a single question about a patient’s willingness to recommend, while this study considered several dimensions of satisfaction.

Larger investments in equipment were associated with better patient-centered care ratings. This has been mentioned by other authors [15]. On the other hand, investing in RNs does not appear to positively influence patient satisfaction. Surprisingly perhaps, availability of staff had lower scores in hospitals where the percentage of RNs was higher. It is possible that this relationship was observed because investments in hiring RN staff left less capacity to hire other staff who could be more responsive to patients’ non-medical needs. While the relationship between availability of RNs and clinical outcomes is well established, the relationship with patient experience is less well understood. Continuing education and satisfaction were not determined to be significantly related. Dansky and colleagues previously found that hours and money spent on continuing education of RNs had a negative influence on patient satisfaction, suggesting that advanced clinical skills does not directly improve patient satisfaction [28]. However, Greenslade and Jimmieson [18] found that promoting a positive service climate within the hospital setting will have a motivating effect on nursing units and contribute to higher patient satisfaction. However, nurses’ task performance did make a difference in patient satisfaction [18]. Finally, hospitals using electronic records had higher scores of satisfaction in patient-centered care and ratings of nurses and doctors, which corroborates prior research [21,29].

### Implications about hospitals performance and quality of care

The results of this study can help orient hospital administrator’s actions to improve patient experience and satisfaction in health care. Enhanced patient satisfaction might ultimately contribute to improvement in health-related outcomes. A recent systematic review by Doyle et al [30] showed evidence of associations between patient satisfaction and a number of outcomes, including mortality, infections, perceived health status, medication compliance, or ED use. To improve satisfaction ratings, hospitals will have to involve families and patients more in decision-making, particularly with patients that have higher levels of education. On the other hand more vulnerable patients, including those who are less educated or marginalized, might benefit from enhanced education and empowerment initiatives to enble them to better express their needs and to be more aware of inadequate care. Patients with lower ratings may have higher expectations, and it could be relevant for the managers to find ways to assess patients’ expectations in order to better address their needs.

Another means to improve patient satisfaction ratings could be for hospitals to increase the number of non-medical staff and emphasize non-technical interpersonal care training for nurses and physicians. Additionally, it is important to consider factors like nurse satisfaction and work environment, both being associated with increased sick leave, which does have a significant impact on patient satisfaction.

One of the strongest predictors of patient satisfaction was the communication of medication information between providers on referral and transfer. This may be a more general indicator of communication within the hospital and suggests that better information exchange among providers is one of the most important determinants of patient satisfaction.

Pain management appears to be an area that is difficult to understand: this dimension showed the lowest variability at both patient and hospital levels (null model statistics in Table 4); while 32% of hospital-level variance was explained, only 6% of individual level variance was explained by the model. This dimension of satisfaction also showed some of the lowest correlations with other dimensions, which confirms that it is a very distinct area requiring specific attention.

The strong association between adverse events and patient satisfaction highlights one dimension, clinical quality improvement, which may improve health outcomes of patients and patient satisfaction scores, and could reduce costs associated with managing adverse events. This suggests an opportune area for quality improvement focus.

The dimensions of patient satisfaction identified in this study could be useful to examine issues related to satisfaction in hospital reports. These dimensions are not the same as those identified by Picker but reflect the ways that patients experience and report on their care experience through the survey data. Hospital comparisons and benchmarking on these dimensions should use risk-adjustment factors for non-modifiable patient characteristics.

## Conclusion

This study uses an appropriate methodology to evaluate patient satisfaction, while taking into account the clustering of responses at the hospital level. Hierarchical regression revealed both individual patient and organizational determinants for understanding customer satisfaction and distinguished between sources of variation. It also elucidated the fact that most variability in patient satisfaction is at the patient rather than the hospital level. Still, hospitals must optimize the impact that they can have. Hierarchical analyses are increasingly used in health services research but have not been widely adopted for the study of satisfaction in health organizations. The findings from this study highlight opportunities to improve patients’ experiences in health care, for example, patient-centered care and pain management, which showed some of lowest average ratings. Significant variations between hospitals in all satisfaction domains were also observed, highlighting the opportunity for improved performance among low-performers.

The analysis included more than 30,000 individuals from 68 hospitals. Nonetheless, a modest response rate of 42% and the lack of data completeness, particularly among the small hospitals, might have introduced some bias into our results. However, this sample size made a powerful analysis possible and a rigorous methodology was employed to both measure satisfaction and analyze its determinants. Moreover, the cross-sectional data used for this study make it challenging to establish with certitude the directionality and validity of the causal links. Data are also old but the associations found represent structural relationships and the structures of the health care system are similar. Not taken into account were the individual clinicians’ characteristics or patient–provider relationships, which could play an important role in patient satisfaction. Even though the variance at the hospital level represents a small portion of the total variance in patient satisfaction and the models explain a substantial percentage of this variance, better results may have been reached if it had been possible to include more organizational indicators. For example, characteristics of work environment were not covered. In addition, the measures used had some limitations and did not fully capture all concepts at the hospital level. For example, the measure of staff roles indicates the variety of roles present but does not specify if and how staff work together toward a more integrated patient care.

However, these findings contribute to a better understanding of patient satisfaction by identifying relevant and specific areas of satisfaction with care. The importance of individual characteristics that impact satisfaction and should be considered to identify patients with high opportunities for improved care were considered. Key organizational characteristics that contribute to better patient experience were identified. Initiatives focusing on patients are important for patient satisfaction and for improving quality of care were specifically demonstrated.

We undertook a sophisticated analysis of the factors of patient satisfaction within domains supported by the survey data and evaluated the impact of organizational factors that could be related to these domains. This approach and our findings are robust and most useful and actionable by hospital providers. However, the development and rigorous evaluation of an overall score based on the broad content of the survey tool might be helpful and useful for future research using the satisfaction tool.

## Competing interests

Dr. Wodchis is a research advisor to NRCC (formerly NRC Picker Canada).

## Authors’ contributions

Both authors have made substantial contributions to the conception and design of the study. AJK has performed the statistical analyses and both AJK and WPW have been involved in the interpretation of the results. AJK has been involved in drafting the manuscript and WPW has profoundly revised it for important intellectual content. Both authors read and approved the final manuscript.
